# Population health status based on the EQ-5D-Y-3L among adolescents in Sweden: Results by sociodemographic factors and self-reported comorbidity

**DOI:** 10.1007/s11136-018-1985-2

**Published:** 2018-09-08

**Authors:** Mimmi Åström, Carina Persson, Margareta Lindén-Boström, Ola Rolfson, Kristina Burström

**Affiliations:** 1 Health Outcomes and Economic Evaluation Research Group, Stockholm Centre for Healthcare Ethics, Department of Learning, Informatics, Management and Ethics, Karolinska Institutet, Tomtebodavägen 18 A, 171 77 Stockholm, Sweden; 2 Equity and Health Policy Research Group, Department of Public Health Sciences, Karolinska Institutet, Stockholm, Sweden; 3 Health Care Services, Stockholm County Council, Stockholm, Sweden; 4 Department for Sustainable Development, Region Örebro County, Örebro, Sweden; 5 Faculty of Medicine and Health, Örebro University, Örebro, Sweden; 6 Department of Orthopaedics, Institute of Clinical Sciences, the Sahlgrenska Academy, University of Gothenburg, Gothenburg, Sweden; 7 Swedish Hip Arthroplasty Register, Gothenburg, Sweden

**Keywords:** Adolescents, EQ-5D-Y-3L, Functional impairment, General population, Mental distress, Parents’ occupational status

## Abstract

**Purpose:**

The EQ-5D-Y-3L is a generic health-related quality of life (HRQoL) measure developed for youth from 8 years old. The aim of this study is to present population health status, based on the EQ-5D-Y-3L, among adolescents in Sweden, by sex, age, self-reported comorbidity and parents’ occupational status.

**Methods:**

Data were obtained from a cross-sectional total survey among students, aged 13–18 years, in a Swedish County year 2014. The survey included EQ-5D-Y-3L, questions regarding self-reported health, disease, functional impairment and mental distress. Parents’ occupational status was used as a proxy for socio-economic status.

**Results:**

A total of 6574 participants answered all the EQ-5D-Y-3L dimensions (mean age was 15.9 years, same proportion of boys and girls). Girls reported more problems than boys in the dimensions ‘doing usual activities’, ‘having pain or discomfort’ and ‘feeling worried, sad or unhappy’, and lower mean VAS score. Respondents with one or both parents unemployed reported more problems with usual activities, pain/discomfort and in the mood dimension than those with both parents employed. Those with comorbidity had in general more problems in all dimensions and lower mean VAS score. The highest impact on VAS score was found for adolescents who reported that they always felt depressed.

**Conclusions:**

Sex, age, self-reported comorbidity and parents’ occupational status were associated with HRQoL determined by the EQ-5D-Y-3L in the general population of adolescents. The ability of EQ-5D-Y-3L to distinguish adolescents’ health status based on these factors confirms the instrument’s usefulness in assessment of HRQoL and as guidance for prioritization.

**Electronic supplementary material:**

The online version of this article (10.1007/s11136-018-1985-2) contains supplementary material, which is available to authorized users.

## Introduction

Each child has the right to the highest achievable health as well as to access to health care, treatment and rehabilitation [1]. Measuring health status in all ages is important when describing and monitoring health in a population and when evaluating treatment effects and conducting economic evaluation in health care [2–4].

Many health care systems emphasize that health should be measured from the perspective of the patient as a complement to clinical measures [5]. This can be achieved by the use of patient-reported outcome measures (PROMs), which are standardized instruments established to capture the patient’s health from the patient’s own point of view [5, 6]. Several PROMs, which include measure of health-related quality of life (HRQoL), targeting children and adolescents have been developed [2, 4, 7]. HRQoL is a multidimensional description of health and includes physical, psychological, social and emotional dimensions that are influenced by changes in health status and important for a person’s overall well-being [8]. HRQoL can be measured with generic or condition-specific instruments [3].

Using self-reported PROMs in children and adolescents entails several methodological challenges, like the child’s development stage, general cognitive competence and understanding of the concepts of health and illness [7, 9, 10]. Proxy measures, where e.g. parents or health care personnel are asked to report the child’s health on behalf of the child have been used as an alternative to children self-reporting their own health [4, 7]. However, since the concept of HRQoL refers to the individual’s own perception of health status, it is desirable to derive information directly from the individual of interest [10, 11]. It has been observed that children from the age of eight can self-report their health in a meaningful way and for children from 12 years old, self-report is preferred [9, 12]. Nevertheless, each child’s pace of development is unique and a clear age cut-off from when children are able to self-report their health is not possible to state [12].

Collecting HRQoL data from the general population of children and adolescents makes it possible to monitor population health status over time and identify groups within the general population with greater risk of poor health [13]. Furthermore, it enables comparisons of health status of the general population with specific patient groups [13–15]. Population data describe health among the general population, commonly by sex, age and socio-economic status. Population data, also referred to as population norms or population reference data [13], are usually collected through general population health surveys [16]. The adult version of the EQ-5D has been used to measure health status in many countries and regions and population data have been established based on the instrument [13, 17, 18]. There are health surveys targeting children and adolescents, but those usually do not include self-reported HRQoL instruments.

The EQ-5D-Y-3L is a generic HRQoL instrument developed by modifying the language and layout of the adult version EQ-5D-3L to make the instrument suitable for children from 8 years old [19, 20]. The EQ-5D-Y-3L is translated to over 40 languages and offers a range of modes of administration, such as self-completion paper and pencil version and versions for tablets and smartphones [21]. The EQ-5D-Y-3L has been tested in terms of feasibility, validity and reliability in general populations of children and adolescents [22, 23]. In clinical studies, the EQ-5D-Y-3L has been validated for a number of health conditions among children and adolescents, such as cystic fibrosis, functional disabilities, asthma and among acutely ill children [24–27]. Population health status among children aged between 7 and 12 years old has been investigated with the EQ-5D-Y-3L instrument [23, 28, 29].

To the best of our knowledge, no population data exist for the general population of adolescents based on the EQ-5D-Y-3L instrument. The aim of this study is to present population health status, based on the EQ-5D-Y-3L, among adolescents in Sweden, by sex, age, self-reported comorbidity and parents’ occupational status.

## Methods

### Study design

Data were obtained from the general population survey Life & Health—young people conducted in year 2014 among Swedish adolescents in Örebro County. The self-administered paper and pencil survey was distributed to all adolescents in grade seven and nine in compulsory school, and in the second year of upper secondary school. The purpose of the survey was to investigate adolescents living conditions, health-related behaviours and health, results from the survey are described elsewhere [30]. The survey was distributed in two versions, one to adolescents in grade seven and one to adolescents in grade nine and in the second year of upper secondary school. Whereas a majority of questions were common between surveys, questions regarding e.g. sexual behaviours and illicit drugs were only asked to adolescents in school year nine and in the second year of upper secondary school.

Teachers or principals at each school informed the pupils about the survey and invited them to answer the survey anonymously in the classroom during school hours. Teachers informed that participation was voluntary that they could withdraw from participation, and that collected data could not be traced to the individual. In the survey, there was written information regarding participation. After completion, participants were asked to put the survey in an envelope and seal it. Prior to the data collection, parents/guardians were informed about the purpose of the survey that participation was voluntary and that they could withdraw their adolescent from participation. Ethical approval was granted by the Regional Ethical Review Board in Uppsala, Sweden (Dnr: 2013/459).

### Measures

The survey consisted of 62 questions for adolescents in school year seven, and 86 questions for adolescents in school year nine and in the second year of upper secondary school. The survey covered questions regarding health, health-related behaviours and living conditions, e.g. questions on socio-demographics, self-rated health (SRH), parents’ occupational status, self-reported disease and functional impairment. In year 2014, the survey included, for the first time, the Swedish version of the EQ-5D-Y-3L instrument.

### EQ-5D-Y-3L

The EQ-5D-Y-3L consists of a descriptive system with five dimensions and a visual analogue scale (VAS), where the respondent is asked to self-report his or her health status today [19, 20]. EQ-5D-Y-3L consists of the following dimensions: ‘mobility’, ‘looking after myself’, ‘doing usual activities’, ‘having pain or discomfort’ and ‘feeling worried, sad or unhappy’. Each dimension has three severity levels: ‘no’ problems, ‘some’ problems and ‘a lot of’ problems. A total of 243 (3^5^) unique health profiles can be derived from the dimensions in combination with the severity levels. For the VAS, the respondent is asked to rate their overall health status between 100 (the best health you can imagine) and 0 (the worst health you can imagine) [19].

### Self-rated health

SRH is a single question frequently used in surveys to assess respondent’s self-reported health [31, 32]. The SRH question was phrased ‘How is your overall health?’ The response options were as follows: ‘very good, good, neither good nor bad, bad, very bad’.

### Parents’ occupational status

In the absence of information on the socio-economic status of parents, parents’ occupational status was used as a proxy. Parents’ occupational status was assessed by a multiple-choice question regarding the respondent’s father’s and mother’s occupational status. Socio-economic status based on adolescents’ reports of parents’ occupational status has been used earlier [33]. In this survey, the questions were framed ‘What does your father and mother do?’ The response options were as follows: ‘working, on sick leave/disability pension, unemployed, studying, on parental leave, other, do not know’. Respondents who chose more than one response option or who did not answer the questions were excluded (*n* = 1520). Father’s and mother’s occupational status were combined into parents’ occupational status (both parents working vs. one or both parents being unemployed).

### Self-reported disease

Self-reported disease was assessed by the multiple-choice question ‘Do you have any of the following diseases? Asthma, allergic eyes or nasal symptoms, food allergy, nickel allergy, eczema, other skin disease, diabetes, or/and epilepsy?’ The answers were dichotomized into yes (mild and severe disease) and no (not having the disease) in the analysis.

### Self-reported functional impairment

Self-reported functional impairment was assessed by the multiple-choice question ‘Do you have any of the following functional impairments? Hearing loss, vision loss, physical impairment, reading- and writing disabilities or dyslexia, Attention Deficit Hyperactivity Disorder (ADHD) (Asperger, Tourette or similar) or/and other functional impairment?’ The answers were dichotomized into yes (mild and severe functional impairment) and no (not having the functional impairment) in the analysis.

### Body mass index

Body mass index (BMI) was calculated from self-reported height and weight of the individual. BMI was calculated by dividing body weight in kilograms by height in meters squared (kg/m^2^) for each respondent. Cut-off points were for underweight < 18.5, normal weight ≥ 18.5 < 25, overweight ≥ 25 < 30 and obesity ≥ 30 [34].

### Mental distress

Mental distress was assessed by two questions ‘During the past 3 months, how often have you felt stressed?’ and ‘During the past 3 months, how often have you felt depressed?’ The response options were as follows: ‘never, rarely, sometimes, often, always’. The answers were dichotomized into yes (always or often) and no (never, rarely or sometimes) in the descriptive analysis.

### Data analyses

Inclusion criterion was being aged 13–18 years at the end of year 2014 i.e. born between the year 1996 and 2001. Respondents who did not provide an answer about sex or age were excluded. Regarding the EQ-5D-Y-3L, a complete case analysis was chosen; hence, respondents with missing values on any of the dimensions were deleted. The complete cases analysis is recommended when the proportion of missing values is small, around < 5% [35].

The sample was divided into three age groups: 13–14 years, 15–16 years and 17–18 years. Calculation of the proportion of adolescents reporting ‘no’, ‘some’ and ‘a lot of’ problems in each EQ-5D-Y-3L dimension was assessed by sex, age group, parents’ occupational status, disease, functional impairment, mental distress, SRH and BMI. To test for statistical significant differences between groups in proportion of reported problems, the Chi-square test or the Fisher’s Exact test was used. Prior to the significance test, the severity levels ‘some’ problems and ‘a lot of’ problems were combined into ‘any’ problems. The Mann–Whitney *U* test was used to test for statistical significant differences in mean VAS scores between groups [36]. Multiple logistic regression analysis was used to investigate associations between reported problems in the EQ-5D-Y-3L dimensions and respondent’s sex, age and parent’s occupational status. The results are presented as odds ratio (OR). Multiple linear regression was used to investigate the association between mean VAS score and sex, age group, parents’ occupational status, disease, functional impairment, mental distress and BMI. A 5% significance level was used and analyses were performed in SPSS 23 [37].

## Results

### Response rate

In total, 7399 pupils answered the survey and the response rate was 79.7%. Respondents with missing or ambiguous answers for sex (1.6%), age (0.9%) and not fulfilling the inclusion criteria of age were excluded, which resulted in 6805 respondents. Those with missing answers on one or more (*n* = 231, 3.4%) of the EQ-5D-Y-3L dimensions (‘mobility’ 0.9%; ‘looking after myself’ 1.4%; ‘doing usual activities’ 1.4%; ‘having pain or discomfort’ 1.7%; ‘feeling worried, sad or unhappy’ 1.8%) were excluded. The final sample for analysis consists of 6574 participants. In the final sample, 106 (1.6%) participants had a missing VAS score.

### Characteristics of study participants

The mean age was 15.9 years and boys comprised half of the sample. A majority of the participants reported that both their parents were working (73.1%). The most commonly self-reported disease and functional impairment were allergic eyes or nasal symptoms (21.5%), and reading- and writing disabilities or dyslexia (7.3%). Among the respondents, 31.8% reported often or always felt stressed, and 14.0% reported often or always felt depressed, during the past 3 months (Table 1).

**Table 1:** Characteristics of study participants (*n* = 6574)

	%	*n*
Age years mean (SD)	15.9	(1.6)
Age groups (years)		
13–14	34.3	2252
15–16	34.3	2254
17–18	31.5	2068
Sex		
Boys	50.6	3324
Girls	49.4	3250
Socio-economic status		
Both parents work	73.1	4805
One or both parents unemployed	3.8	249
Missing	23.1	1520
Self-rated health		
Very good	39.6	2601
Good	43.7	2870
Neither good or bad	11.9	785
Bad	3.1	203
Very bad	1.1	70
Missing	0.7	45
Self-reported disease		
Allergic eyes/nasal symptoms	21.5	1415
Eczema	11.8	775
Asthma	11.4	749
Nickel allergy	9.0	592
Food allergy	7.2	475
Other skin disease	3.9	257
Diabetes	0.8	52
Epilepsy	0.6	38
Self-reported functional impairment		
Reading- and writing disabilities or dyslexia	7.3	481
Hearing loss	6.2	409
ADHD, Asperger, Tourette or similar	4.5	299
Vision loss	3.8	251
Physical disability	2.3	150
Other functional disability	2.2	142
Self-reported BMI		
Underweight	8.4	555
Normal weight	66.4	4366
Overweight	12.4	818
Obesity	3.0	195
Missing	9.7	640
Self-reported stress^a^		
Never stressed	10.2	673
Rarely stressed	23.6	1552
Sometimes stressed	33.7	2217
Often stressed	26.2	1720
Always stressed	5.6	370
Missing	0.6	42
Self-reported depression^a^		
Never depressed	26.9	1771
Rarely depressed	32.3	2125
Sometimes depressed	24.3	1595
Often depressed	12.0	787
Always depressed	2.0	132
Missing	2.5	164

^a^During the past 3 months

### Health profiles

There were in total 94 unique health profiles in the sample. Most frequently reported (44.9%) was the health profile 11111 (i.e. no problems in any of the EQ-5D-Y-3L dimensions). The health profile 33333 (i.e. a lot of problems in all dimensions) was reported by two respondents (Online Appendix Table 1).

### EQ-5D-Y-3L by sex and age group

In total, most problems were reported in the dimensions ‘having pain or discomfort’ and ‘feeling worried, sad or unhappy’. Differences in proportion of reported problems between boys and girls were found, girls reported more problems in the dimensions ‘doing usual activities’, ‘having pain or discomfort’ and ‘feeling worried, sad or unhappy’. Highest proportion of reported problems for girls was in the mood dimension and for boys with pain and discomfort. Girls reported lower mean VAS score (71.8) than did boys (78.9) (Table 2).

**Table 2:**
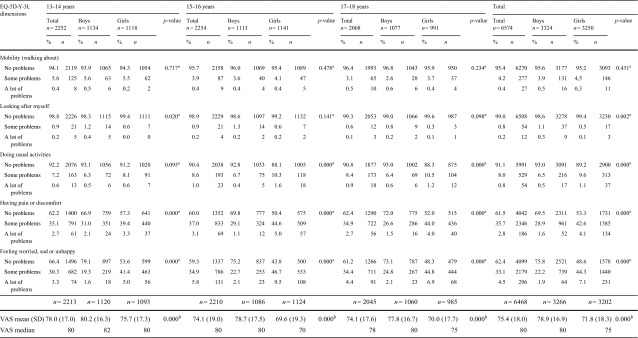
Distribution (%, *n*) of reported problems in the EQ-5D-Y-3L dimensions, VAS mean value (SD) and VAS median, by age group and sex

Age in years; 13 years (*n* = 22), 14 years (*n* = 2230), 15 years (*n* = 118), 16 years (*n* = 2136), 17 years (*n* = 102), 18 years (*n* = 1966). Some and a lot of problems were collapsed into any problems when testing for significant differences

^a^ *p*-value by Chi-square test

^b^ *p*-value by Mann–Whitney U test

The youngest age group, 13–14 years, were those who reported most problems in the ‘mobility’ dimension. Respondents aged 15–16 years reported most problems in the dimensions ‘doing usual activities’, ‘having pain or discomfort’ and ‘feeling worried, sad or unhappy’, compared to the other age groups. The youngest age group, 13–14 years, reported the highest mean VAS score (78.0), compared to the older age groups (Table 2).

Girls in all age groups reported most problems in the mood dimension while boys, in all age groups, reported most problems with pain/discomfort. In the youngest age group, 13–14 years, girls reported more problems than boys did with pain/discomfort and in the mood dimension. In the age groups 15–16 years and 17–18 years, girls reported more problems than boys in the dimensions ‘doing usual activities’, ‘having pain or discomfort’ and ‘feeling worried, sad or unhappy’. Girls in all age groups reported lower mean VAS scores than boys, in particular in the age group 15–16 years (69.6) (Table 2).

### EQ-5D-Y-3L by parents’ occupational status

Respondents with one or both parents being unemployed compared to those with both parents working, reported more problems with usual activities, pain/discomfort and in the mood dimension. Respondents with one or both parents being unemployed reported lower mean VAS score (Table 3).

**Table 3:** Distribution (%, *n*) of reported problems in the EQ-5D-Y-3L dimensions, VAS mean value (SD) and VAS median by parents’ occupational status

EQ-5D-Y-3L dimensions	One or both parents unemployed		Both parents work		*p*-value
	*n* = 249		*n* = 4805		
	%	*n*	%	*n*	
Mobility (walking about)					
Some problems	3.2	8	3.6	175	0.527^a^
A lot of problems	0.0	0	0.4	18	
Looking after myself					
Some problems	1.6	4	0.7	35	0.286^b^
A lot of problems	0.0	0	0.1	7	
Doing usual activities					
Some problems	10.0	25	7.1	343	0.030^a^
A lot of problems	1.6	4	0.7	33	
Having pain or discomfort					
Some problems	44.2	110	34.8	1670	0.001^a^
A lot of problems	3.2	8	2.5	121	
Feeling worried, sad or unhappy					
Some problems	40.6	101	31.4	1,51	0.000^a^
A lot of problems	6.4	16	3.8	181	

	*n* = 244		*n* = 4737		
VAS mean (SD)	72.3 (18.8)		76.1 (17.3)		0.000^c^
VAS median	75		80		

Some and a lot of problems were collapsed into any problems when testing for significant differences

^a^ *p*-value by Chi-square test

^b^ *p*-value by Fisher’s Exact Test

^c^ *p*-value by Mann–Whitney U test

### EQ-5D-Y-3L by self-reported disease, functional impairment and mental distress

Respondents with asthma, allergic eyes/nasal symptoms, food allergy, nickel allergy and skin disease reported more problems in all EQ-5D-Y-3L dimensions except in the dimension ‘looking after myself’, and lower mean VAS score, than those not reporting that specific disease. No differences in reported problems were found for respondents with diabetes compared to those not reporting diabetes. Respondents with epilepsy had most problems across all dimensions, except for the mood dimension, and had the lowest mean VAS score (65.4) (Table 4).

**Table 4:**
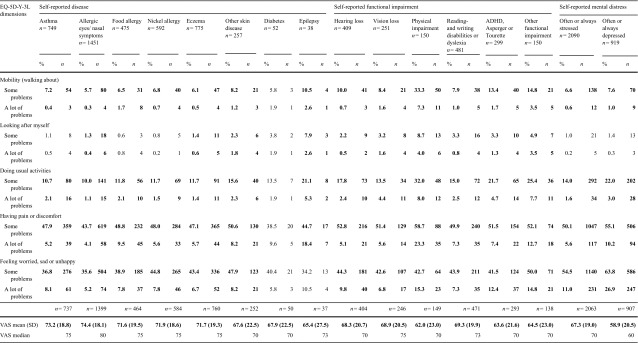
Distribution (%, *n*) of reported problems in the EQ-5D-Y-3L dimensions, VAS mean value (SD) and VAS median, by self-reported disease, functional impairment and mental distress

Bold—statistical significant more problems reported by those with the comorbidity compared to those without that particular comorbidity (some and a lot of problems were collapsed into any problems when testing for significant differences)

Respondents with impairment reported more problems across all EQ-5D-Y-3L dimensions and lower mean VAS score than those not reporting that specific impairment. Among respondents with a physical impairment, 82.0% had problems with pain/discomfort; further, this group reported most problems across all dimensions, except the mood dimension, and the lowest mean VAS score (62.0) (Table 4).

Respondents, who often or always felt stressed or depressed during the past 3 months, reported more problems in all EQ-5D-Y-3L dimensions except in the dimensions ‘looking after myself’, and lower VAS score compared to those reported mental distress never, rarely or sometimes. Among those reporting always felt depressed, 90.7% had problems in the mood dimension (Table 4).

### EQ-5D-Y-3L by self-rated health

There was a gradient in reported problems in the EQ-5D-Y-3L dimensions from ‘very good’ to ‘very bad’ SRH (Online Appendix Fig. 1). Respondents who answered ‘very bad’ to the SRH question reported the lowest mean VAS score (Online Appendix Fig. 2).

### EQ-5D-Y-3L by body mass index

Adolescents who had a BMI ≥ 30 and thus classified as obese, reported most problems across all EQ-5D-Y-3L dimensions. Among respondents classified as obese, the highest proportion of reported problems were with pain/discomfort and in the mood dimension. Respondents classified as obese also reported the lowest mean VAS score (66.9) (Online Appendix Table 2).

### Regression analyses

Controlling for age and parents’ occupational status, girls were more likely than boys to report ‘some’ or ‘a lot’ problems in the dimensions ‘doing usual activities’, ‘having pain or discomfort’ and ‘feeling worried, sad or unhappy’ (Table 5). For girls compared to boys, the highest odds (OR 3.44) of reporting any problems was found for the mood dimension. Regarding age, adolescents in the age group 15–16 years were more likely to have problems in the mood dimension and less likely to report problems in the ‘mobility’ dimension, compared to the youngest age group. Controlling for age and sex, adolescents with one or both parents being unemployed were more likely to report problems with usual activities, pain/discomfort and in the mood dimension (Table 5).

**Table 5:** ORs (95% confidence intervals) for reporting some or a lot of problems on the EQ-5D-Y-3L dimensions controlled for sex, age and parents’ occupational status

	Mobility (walking about)			Looking after myself			Doing usual activities			Having pain or discomfort			Feeling worried, sad or unhappy		
	OR	95% CI	*p*-value	OR	95% CI	*p*-value	OR	95% CI	*p*-value	OR	95% CI	*p*-value	OR	95% CI	*p*-value
Sex															
Boys	Reference														
Girls	1.04	(0.78–1.37)	0.807	0.55	(0.30–1.02)	0.057	**1.53**	**(1.24–1.88)**	**0.000**	**1.97**	**(1.75–2.21)**	**0.000**	**3.44**	**(3.04–3.88)**	**0.000**
Age															
13–14	Reference														
15–16	**0.57**	**(0.40–0.81)**	**0.002**	0.66	(0.32–1.35)	0.253	1.30	(1.00–1.68)	0.051	1.02	(0.89–1.18)	0.747	**1.27**	**(1.09–1.47)**	**0.002**
17–18	0.80	(0.55–1.16)	0.237	0.83	(0.39–1.77)	0.627	0.92	(0.72–1.17)	0.489	0.88	(0.77–1.02)	0.087	0.97	(0.84–1.12)	0.688
Parents’ occupational status															
Both parents employed	Reference														
One or both parents unemployed	0.79	(0.38–1.62)	0.518	1.86	(0.66–5.23)	0.240	**1.55**	**(1.03–2.31)**	**0.034**	**1.52**	**(1.17–1.97)**	0.002	**1.68**	**(1.28–2.20)**	**0.000**

Some and a lot of problems were collapsed into any problems

Bold: statistically significant < 0.05

Variation of VAS score by sex, age, self-reported disease, functional impairment, mental distress, parents’ occupational status and BMI is shown in Table 6. Girls reported lower VAS score than boys and the older age groups (15–16 years and 17–18 years) reported lower VAS score than those 13–14 years old (Model 1). Controlling for sex and age, adolescents with one or both parents being unemployed reported lower VAS score (Model 2). After controlling for sex and age, adolescents with underweight, overweight and obesity, had lower VAS score compared to those with normal weight (Model 3). These differences remained when also controlling for parents’ occupational status (Model 4). After controlling for sex and age, adolescents who self-reported other skin disease, diabetes, epilepsy, reading or writing disabilities or dyslexia, hearing loss, ADHD, physical impairment, stress or depression, reported lower VAS score compared to those not having the disease or the functional impairment, or never feeling stressed or depressed (Model 5). When also controlling for parents’ occupational status, the association with VAS score remained for all diseases, functional impairments and mental distress, except for respondents with diabetes (Model 6). The highest association with VAS score was found for adolescents who reported feeling depressed “always” for the past 3 months (28.6), controlling for all other factors (Model 7).

**Table 6:** Multiple linear regression analyses on VAS score, controlling for sex, age group, parents’ occupational status, self-reported disease, functional impairment, mental distress and BMI

	Model 1		Model 2		Model 3		Model 4		Model 5		Model 6		Model 7	
	*β*-Estimate	*p*-value	*β*-Estimate	*p*-value	*β*-Estimate	*p*-value	*β*-Estimate	*p*-value	*β*-Estimate	*p*-value	*β*-Estimate	*p*-value	*β*-Estimate	*p*-value
Intercept	**81.49**	< **0.001**	**82.01**	< **0.001**	**83.40**	< **0.001**	**83.66**	< **0.001**	**88.21**	< **0.001**	**88.30**	< **0.001**	**90.30**	< **0.001**
Sex^a^	**− 7.15**	< **0.001**	**− 7.01**	< **0.001**	**− 7.59**	< **0.001**	**− 7.43**	< **0.001**	**− 1.35**	**0.004**	**− 1.09**	**0.036**	**− 1.59**	**0.003**
Age group^b^														
15–16	**− 3.78**	< **0.001**	**− 3.60**	< **0.001**	**− 4.08**	< **0.001**	**− 3.68**	< **0.001**	**− 1.38**	**0.007**	− 1.02	0.071	**− 1.37**	**0.019**
17–18	**− 3.98**	< **0.001**	**− 3.97**	< **0.001**	**− 4.43**	< **0.001**	**− 4.13**	< **0.001**	− 0.70	0.185	− 0.91	0.117	**− 1.59**	**0.020**
Parents’ occupational status^c^														
One or both parents unemployed			**− 3.65**	**0.001**			**− 3.48**	**0.002**			**− 2.87**	**0.008**	**− 2.589**	**0.020**
Self-reported disease and functional impairment^d^														
Allergic eyes/nasal symptoms									0.29	0.592	− 0.31	0.603	− 0.53	0.381
Eczema									− 0.80	0.242	− 0.64	0.390	− 1.07	0.155
Asthma									0.43	0.541	0.26	0.736	0.21	0.792
Nickel allergy									0.21	0.785	− 1.15	0.166	− 1.22	0.149
Food allergy									− 0.62	0.451	− 1.65	0.077	− 1.54	0.094
Other skin disease									**− 3.16**	**0.004**	**− 2.65**	**0.033**	**− 2.75**	**0.027**
Diabetes									**− 4.45**	**0.028**	− 2.74	0.321	− 2.28	0.402
Epilepsy									**− 9.16**	**0.002**	**− 8.07**	**0.013**	**− 8.55**	**0.009**
Reading/writing disabilities or dyslexia									**− 2.27**	**0.007**	**− 2.14**	**0.026**	− 1.60	0.112
Hearing loss									**− 2.59**	**0.004**	**− 2.34**	**0.019**	− 1.23	0.238
ADHD, Asperger or Tourette									**− 6.35**	< **0.001**	**− 6.64**	< **0.001**	**− 6.88**	< **0.001**
Vision loss									− 1.59	0.158	− 0.39	0.773	1.41	0.311
Physical disability									**− 8.21**	< **0.001**	**− 7.99**	< **0.001**	**− 7.39**	< **0.001**
Other functional disability									− 2.60	0.075	− 2.52	0.143	− 3.08	0.091
Self-reported mental distress^e^														
Rarely stressed									− 1.43	0.074	− 1.63	0.066	**− 1.95**	**0.034**
Sometimes stressed									**− 3.17**	< **0.001**	**− 2.73**	**0.002**	**− 2.93**	**0.002**
Often stressed									**− 6.17**	< **0.001**	**− 5.83**	< **0.001**	**− 5.83**	< **0.001**
Always stressed									**− 8.24**	< **0.001**	**− 8.30**	< **0.001**	**− 8.54**	< **0.001**
Rarely depressed									**− 4.69**	< **0.001**	**− 4.31**	< **0.001**	**− 4.51**	< **0.001**
Sometimes depressed									**− 9.24**	< **0.001**	**− 9.64**	< **0.001**	**− 9.96**	< **0.001**
Often depressed									**− 18.70**	< **0.001**	**− 18.04**	< **0.001**	**− 17.73**	< **0.001**
Always depressed									**− 28.03**	< **0.001**	**− 28.91**	< **0.001**	**− 28.55**	< **0.001**
Self-reported BMI^f^														
Underweight					**− 1.85**	**0.017**	**− 2.12**	**0.015**					**− 2.08**	**0.012**
Overweight					**− 3.80**	< **0.001**	**− 3.84**	< **0.001**					**− 4.37**	< **0.001**
Obesity					**− 10.52**	< **0.001**	**− 11.01**	< **0.001**					**− 10.34**	< **0.001**
* R* ^2^		0.050		0.053		0.071		0.074		0.258		0.258		0.274
Adjusted *R*^2^		0.050		0.053		0.070		0.072		0.254		0.253		0.269

Bold: statistically significant < 0.05

Reference groups: ^a^boys; ^b^age group 13–14 years; ^c^both parents employed; ^d^not reporting the disease/functional impairment; ^e^never stressed/depressed; ^f^normal weight

## Discussion

This is the first study presenting population health status, based on the EQ-5D-Y-3L, among adolescents in Sweden, by sex, age, self-reported comorbidity and parents’ occupational status. The study shows how adolescents in a general population report their subjective HRQoL and how factors such as disease, functional impairment, and mental distress associate with HRQoL. The study identifies girls compared to boys, older age groups compared to younger and those with one or both parents being unemployed, having reduced HRQoL, measured with the EQ-5D-Y-3L.

The fact that girls reported worse health status than did boys has not been found in earlier studies using the EQ-5D-Y-3L [23, 25, 26]. Although, in studies among adults, using the EQ-5D-3L, women commonly report worse health status than men [14, 17]. In the present study, the observed differences in reported problems in the dimensions and in VAS score between boys and girls, remained significant even after controlling for other factors. The high prevalence of reported problems among girls in the mood dimension is alarming and a cause for concern. Especially noticeable in the age group 15–16 years, where nearly 10% reported ‘a lot of’ problems in the mood dimension. A similar finding, with a relatively high prevalence of reported problems in the mood dimension, has been seen among young women in Sweden [17]. This is also in line with previous results among adolescents, where girls have been reporting worse subjective health than boys [38].

Despite that the present study was conducted among a general population of adolescents, more than half of all respondents reported problems in at least one of the EQ-5D-Y-3L dimensions which was higher than observed earlier [29]. The age range of participants in our study could explain these findings, as it is recognized that adolescents go through several life-challenging changes e.g. increased social pressure from peers and detaching from parents, which can affect HRQoL [39]. Differences in both directions, regarding HRQoL measured with the EQ-5D-Y-3L instrument between younger ages have been observed [23, 26] but more studies are needed to investigate differences in HRQoL between early and late adolescence. In line with the findings from the present study, adolescents have reported lower HRQoL than children, using another instrument [40]. It is of importance to stress potential differences in HRQoL between late and early adolescence, when comparing health of specific patient groups with general population data.

The positive association between health and socio-economic status is well known [41]. Children’s health status have shown to be positively associated with household income and the association even more distinct as children grow older into adolescence [42]. How to measure socio-economic status among children and adolescents has been discussed, previously parents’ occupation, income level and educational level have been used as indicators [28, 29]. In the present study, parents’ occupational status reported by the adolescent was used as a proxy for socio-economic status. Adolescents with one or both parents unemployed reported worse health status. This is in line with previous studies, where children from families with the lowest household income showed the lowest VAS index scores, and children with parents with the lowest educational level reported more problems in the EQ-5D-Y-3L dimensions [28, 29]. If adolescents are able to report their parents’ occupational status in a reliable matter can be discussed and might be a limitation of our study. However, good agreement between adolescents’ reports and parents’ self-reports regarding parents occupational status has been showed earlier [33].

Our study shows that self-reported disease, functional impairment and mental distress have a negative association with the prevalence of reported problems in the EQ-5D-Y-3L dimensions. Children and adolescents with health conditions or functional disability have also reported more problems in the dimensions and lower VAS score compared to those with no illness or functional disability [23]. Children and adolescents with a functional disability have also reported more problems compared to a general population sample [25]. Furthermore, in line with the findings from our study, children and adolescents with overweight or obesity have earlier reported more problems in all the dimensions and lower mean VAS score [23].

The overall results from the survey Life & Health—young people are continuously used as a basis for decisions and priorities within the county, as well as for support for school health promotion [30]. To distribute the survey during school hours was successful as it resulted in a high response rate. This was the first year of including the EQ-5D-Y-3L instrument in the survey, and feasibility was indicated by few missing or ambiguous answers across all dimensions and for the VAS. Feasibility of the EQ-5D-Y-3L has been investigated in a similar way in previous studies [22, 23, 26, 29]. Two adolescents reported ‘a lot of’ problems in all the EQ-5D-Y-3L dimensions. As the survey was distributed during school hours, it could be questioned whether adolescents with these amount of problems are able to attend school; however, these adolescents also answered *very bad* to the SRH question.

For the use of HRQoL instruments in economic assessments, there is a need to obtain a value attached to each health state to create a value set [43]. In the long term, it is essential to develop a value set for the EQ-5D-Y-3L to make it possible to use the instrument in economic evaluation of health care. Hence, as for today, there is no available value set for the EQ-5D-Y-3L [22], and it is not recommended to use the value set for the adult version for the EQ-5D-Y-3L [44]. Studies, where adults have valued health states for children and adolescents based on the EQ-5D-Y-3L [45] and where adolescents have valued health states themselves based on other HRQoL measures [46], have been conducted. However, further studies to investigate how potential valuation methods works when used among children and adolescents are warranted.

## Conclusions

The EQ-5D-Y-3L instrument is able to detect lower self-reported HRQoL among certain groups in the general population of adolescents. The results clearly show that sex, age and parents’ occupational status are associated with health status. Furthermore, adolescents who self-report disease, functional impairment and mental distress report worse health status. When planning health interventions for adolescents, these findings can be used as guidance for prioritization, as all children and adolescents have the right to the highest achievable health.

## Electronic supplementary material

Below is the link to the electronic supplementary material.

Supplementary Table 1 (DOCX 15 KB)

Supplementary Table 2 (DOCX 14 KB)

Supplementary Figure 1 (JPG 42 KB)

Supplementary Figure 2 (JPG 33 KB)
