# Supplementary material for: Population health status based on the EQ-5D-Y-3L among adolescents in Sweden: Results by sociodemographic factors and self-reported comorbidity
Source: Qual Life Res. 2018 Sep 8;27(11):2859–71. doi: 10.1007/s11136-018-1985-2 (PMC6208600; doi:10.1007/s11136-018-1985-2)
Supplement: Supplementary file 1 — Supplementary Table 1 (DOCX 15 KB) [file 11136_2018_1985_MOESM1_ESM.docx]

Table 1. Percentage (number) of adolescents by reported EQ-5D-Y-3L health profiles

| EQ-5D-Y-3L health profile | % | n |
| --- | --- | --- |
| 11111 | 44.9 | 2,955 |
| 11122 | 13.7 | 900 |
| 11112 | 13.2 | 867 |
| 11121 | 13.2 | 867 |
| 11222 | 2.1 | 136 |
| 11123 | 1.5 | 96 |
| 11221 | 1.1 | 70 |
| 21121 | 0.9 | 56 |
| 21122 | 0.8 | 50 |
| 11113 | 0.7 | 49 |
| 11212 | 0.7 | 49 |
| 11223 | 0.7 | 45 |
| 11132 | 0.7 | 43 |
| 11211 | 0.7 | 43 |
| 21222 | 0.5 | 35 |
| 11232 | 0.3 | 20 |
| 21111 | 0.3 | 19 |
| 11213 | 0.3 | 18 |
| 11233 | 0.3 | 18 |
| 21221 | 0.3 | 17 |
| 11131 | 0.2 | 14 |
| 11133 | 0.2 | 10 |
| 21132 | 0.2 | 10 |
| 21223 | 0.2 | 10 |
| 11231 | 0.1 | 9 |
| 11333 | 0.1 | 9 |
| 21123 | 0.1 | 9 |
| 11322 | 0.1 | 8 |
| 21112 | 0.1 | 8 |
| 12111 | 0.1 | 7 |
| 21232 | 0.1 | 7 |
| 21233 | 0.1 | 7 |
| 12222 | 0.1 | 6 |
| 21212 | 0.1 | 5 |
| 21231 | 0.1 | 5 |
| 11323 | 0.1 | 4 |
| 12122 | 0.1 | 4 |
| 22221 | 0.1 | 4 |
| 22222 | 0.1 | 4 |
| 31111 | 0.1 | 4 |
| 12112 | 0.0 | 3 |
| 12221 | 0.0 | 3 |
| 21211 | 0.0 | 3 |
| 21322 | 0.0 | 3 |
| 11312 | 0.0 | 2 |
| 12323 | 0.0 | 2 |
| 13111 | 0.0 | 2 |
| 21131 | 0.0 | 2 |
| 21323 | 0.0 | 2 |
| 21331 | 0.0 | 2 |
| 21332 | 0.0 | 2 |
| 21333 | 0.0 | 2 |
| 22232 | 0.0 | 2 |
| 22333 | 0.0 | 2 |
| 31121 | 0.0 | 2 |
| 31133 | 0.0 | 2 |
| 31232 | 0.0 | 2 |
| 31332 | 0.0 | 2 |
| 33333 | 0.0 | 2 |
| 11313 | 0.0 | 1 |
| 11321 | 0.0 | 1 |
| 11332 | 0.0 | 1 |
| 12121 | 0.0 | 1 |
| 12132 | 0.0 | 1 |
| 12133 | 0.0 | 1 |
| 12212 | 0.0 | 1 |
| 12223 | 0.0 | 1 |
| 12233 | 0.0 | 1 |
| 12321 | 0.0 | 1 |
| 13211 | 0.0 | 1 |
| 21213 | 0.0 | 1 |
| 22111 | 0.0 | 1 |
| 22121 | 0.0 | 1 |
| 22211 | 0.0 | 1 |
| 22231 | 0.0 | 1 |
| 22322 | 0.0 | 1 |
| 22331 | 0.0 | 1 |
| 22332 | 0.0 | 1 |
| 23132 | 0.0 | 1 |
| 23221 | 0.0 | 1 |
| 23323 | 0.0 | 1 |
| 31112 | 0.0 | 1 |
| 31132 | 0.0 | 1 |
| 31221 | 0.0 | 1 |
| 31233 | 0.0 | 1 |
| 31322 | 0.0 | 1 |
| 31333 | 0.0 | 1 |
| 32121 | 0.0 | 1 |
| 32221 | 0.0 | 1 |
| 32332 | 0.0 | 1 |
| 33111 | 0.0 | 1 |
| 33131 | 0.0 | 1 |
| 33132 | 0.0 | 1 |
| 33323 | 0.0 | 1 |
