# Supplementary material for: Population health status based on the EQ-5D-Y-3L among adolescents in Sweden: Results by sociodemographic factors and self-reported comorbidity
Source: Qual Life Res. 2018 Sep 8;27(11):2859–71. doi: 10.1007/s11136-018-1985-2 (PMC6208600; doi:10.1007/s11136-018-1985-2)
Supplement: Supplementary file 2 — Supplementary Table 2 (DOCX 14 KB) [file 11136_2018_1985_MOESM2_ESM.docx]

Table 2. Distribution (%,n) of reported problems in the EQ-5D-Y-3L dimensions, VAS mean value (SD) and VAS median by Body Mass Index (BMI)

|  | Underweight | | Normal weight | | Overweight | | Obesity | |
| --- | --- | --- | --- | --- | --- | --- | --- | --- |
|  | n=555 | | n=4,366 | | n=818 | | n=195 | |
| EQ-5D-Y-3L dimensions | % | n | % | n | % | n | % | n |
| Mobility (walking about) |  |  |  |  |  |  |  |  |
| Some problems | 3.2 | 18 | 4.1 | 179 | 5.0 | 41 | 9.2 | 18 |
| A lot of problems | 0.4 | 2 | 0.2 | 10 | 0.5 | 4 | 1.5 | 3 |
| Looking after myself |  |  |  |  |  |  |  |  |
| Some problems | 0.9 | 5 | 0.6 | 26 | 1.1 | 9 | 1.5 | 3 |
| A lot of problems | 0.2 | 1 | 0.1 | 6 | 0.1 | 1 | 0.5 | 1 |
| Doing usual activities |  |  |  |  |  |  |  |  |
| Some problems | 8.1 | 45 | 7.6 | 332 | 7.8 | 64 | 12.3 | 24 |
| A lot of problems | 1.4 | 8 | 0.8 | 33 | 0.1 | 1 | 2.1 | 4 |
| Having pain or discomfort |  |  |  |  |  |  |  |  |
| Some problems | 36.2 | 201 | 35.2 | 1,537 | 36.7 | 300 | 45.1 | 88 |
| A lot of problems | 3.1 | 17 | 2.7 | 119 | 2.6 | 21 | 2.6 | 5 |
| Feeling worried, sad or unhappy |  |  |  |  |  |  |  |  |
| Some problems | 37.3 | 207 | 32.6 | 1,425 | 32.2 | 263 | 37.9 | 74 |
| A lot of problems | 5.2 | 29 | 4.0 | 175 | 4.3 | 35 | 6.7 | 13 |
|  | n=550 | | n=4,312 | | n=803 | | n=193 | |
| VAS mean (SD) | 74.2 (19.0) | | 76.7 (17.1) | | 74.0 (18.5) | | 66.9 (21.3) | |
| VAS median | 78 | | 80 | | 75 | | 70 | |
