# Supplementary figures and images for: Population health status based on the EQ-5D-Y-3L among adolescents in Sweden: Results by sociodemographic factors and self-reported comorbidity
Source: Qual Life Res. 2018 Sep 8;27(11):2859–71. doi: 10.1007/s11136-018-1985-2 (PMC6208600; doi:10.1007/s11136-018-1985-2)

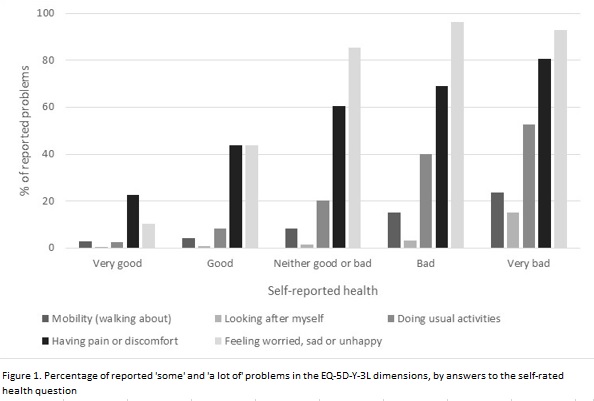

Supplement: Supplementary file 3 — Supplementary Figure 1 (JPG 42 KB) [file 11136_2018_1985_MOESM3_ESM.jpg]

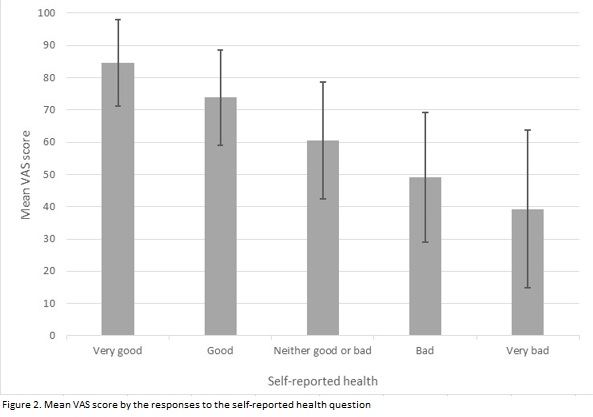

Supplement: Supplementary file 4 — Supplementary Figure 2 (JPG 33 KB) [file 11136_2018_1985_MOESM4_ESM.jpg]
